# Daily blood pressure variability in relation to neurological functional outcomes after acute ischemic stroke

**DOI:** 10.3389/fneur.2022.958166

**Published:** 2023-01-09

**Authors:** Yuan Zhu, Minghua Wu, Huihui Wang, Yawei Zheng, Siqi Zhang, Xintong Wang, Shana Wang, Zhuyuan Fang

**Affiliations:** ^1^Affiliated Hospital of Nanjing University of Chinese Medicine, Jiangsu Province Hospital of Chinese Medicine, Nanjing, China; ^2^Department of Clinical Medicine, Hangzhou Medical College, Hangzhou, China

**Keywords:** acute ischemic stroke, blood pressure variability, neurological functional outcome, hypertension, risk factors

## Abstract

**Background:**

Prior research has shown inconclusive findings regarding the relationship between blood pressure variability (BPV) in acute ischemic stroke (AIS) and functional outcomes. Most research has examined the connection between short-term BPV during the early 24–72 h after the occurrence of ischemic stroke and functional prognosis. We sought to determine the relationship between daily BPV at 7 days of commencement and functional outcomes during the 3 months following AIS.

**Methods:**

Altogether, 633 patients with AIS admitted within 72 h of commencement were enrolled. AIS was defined as the time from the onset of symptoms to 7 days. Throughout this period, blood pressure (BP) was recorded twice daily (casual BP cuffs). The daily BPV, with standard deviation (SD) and coefficient of variation (CV), was calculated and matched to the functional results. The adverse outcome was characterized as a modified Rankin scale (mRS)≥3, which comprised the recurrence of stroke, clinical intracranial bleeding, and death.

**Results:**

In total, 633 participants were included, and the incidence of adverse outcomes was 14.06% (89/633). There was a significant positive correlation between daily BPV and adverse outcomes but not between mean BP and risk. Smooth curve fitting revealed a U-shaped connection between the mean BP and adverse clinical outcomes. Multivariable logistic regression analysis showed an independent correlation between daily BPV and an adverse outcome in the top vs. bottom quartile of systolic BPV (odds ratio [OR] = 2.4, 95% confidence interval [CI]: 1.17–4.96, *P* = 0.018 for SD; OR = 2.4, 95% CI: 1.17–4.93, *P* = 0.017 for CV) during a 3-month follow-up period. Identical results have been reported for diastolic BPV.

**Conclusion:**

Irrespective of BP level, elevated daily systolic BPV and diastolic BPV in AIS were associated with an increased risk of adverse outcomes within 3 months. We also discovered a U-shaped association between the mean BP and adverse clinical outcomes. These findings suggested that BPV should be a risk factor for adverse outcomes after ischemic stroke, which provided new insight into BP management strategy.

## 1. Introduction

Stroke has a high incidence of morbidity, impairment, fatality, and relapse. Accordingly, it severely affects both public health and well-being. Hence, stroke is the main cause of mortality in Chinese people (1). Hypertension is the most frequent adjustable risk indicator for ischemic stroke (2). The ideal blood pressure (BP) control during the acute phase of acute ischemic stroke (AIS) remains undeveloped and debatable (3). Multiple investigations have demonstrated that decreasing BP in patients with AIS has no impact on clinical results (4, 5). Recent research has indicated that in the earliest stages of acute stroke, BP fluctuation may be more relevant than BP level (6).

Rare data exist regarding the impact of BP variability (BPV) on functional outcomes following AIS. Most studies have investigated the connection between short-term BPV in the early 24–72 h following the occurrence of an ischemic stroke and its functional outcome (7–10). The acute stage of ischemic stroke is described as the first 7 days following the appearance of symptoms. In the first acute stage of an ischemic stroke, the patient may experience reactive hypertension and diminished cerebral blood flow (11), and increased BP will become stable within the first 4–7 days after the onset of the stroke (12). However, information on the association between mid-term BPV and outcomes in AIS is insufficient. This study aimed to analyze the connection between daily BPV within 7 days of commencement and functional outcomes within 3 months in individuals with AIS.

## 2. Materials and methods

### 2.1. Patient selection

A retrospective, single-center, observational investigation was conducted.

**Figure 1:**
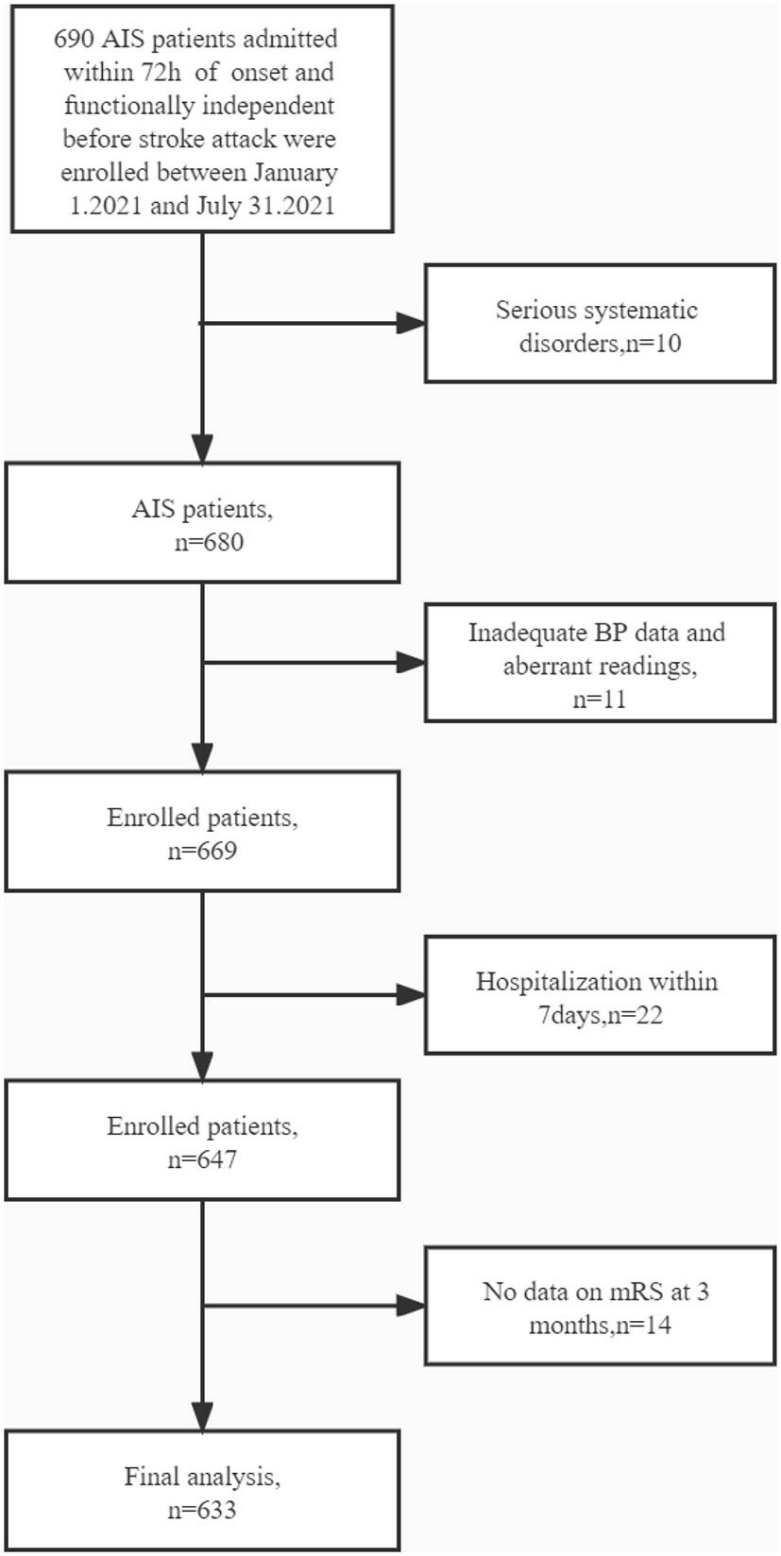
Flowchart demonstrating patient selection.

The ethics committee of Jiangsu Province Hospital of Traditional Chinese Medicine approved the experiments. Between 1 January 2021 and 31 July 2021, the neurological department of the Jiangsu Province Hospital of Traditional Chinese Medicine admitted 690 individuals who were diagnosed with AIS and functionally independent before a stroke attack. The inclusion criteria were as follows: age ≥18 years; management according to the Chinese recommendations for diagnosing and treating AIS (13, 14); brought to the hospital within 72 h of onset; and admission to the hospital for >7 days. Patients who fulfilled the following criteria were excluded from the investigation: with serious systematic disorders or a life probability of <90 days, such as advanced stages of heart failure or cancers, and insufficient measurement of BP and aberrant readings for either systolic blood pressure (SBP) or diastolic blood pressure (DBP), where an aberrant SBP was described as >260 or <70 mmHg, and an abnormal DBP was described as >150 or <40 mmHg (Figure 1).

### 2.2. Demographic and clinical assessment

At the time of registration, patient profiles (such as age and sex), clinical characteristics including early neurological deterioration (END), intravenous thrombolysis, mechanical thrombectomy, and risk indicators such as hypertension (HT), diabetes mellitus (DM), atrial fibrillation (AF), hyperlipidemia, hyperhomocysteinemia, smoking, and alcohol drinking were noted. The patients' medical history records on HT, DM, AF, coronary artery sickness, stroke, and transient ischemic attack were analyzed. Laboratory results, including fasting plasma glucose (FPG) when admitted, low-density lipoprotein (LDL) high-density lipoprotein (HDL), and neutrophil count, were recorded. Hypertension was classified into two classes: not using antihypertensive medications, but with a BP of 140/90 mmHg on repeated measurements, or existing intake of antihypertensive medications (15). DM was defined as FPG ≥ 126 mg/dl, a positive result in the ≥75 g oral glucose tolerance testing, or present therapy with oral hypoglycemic treatment or insulin to control blood glucose. END was defined as any deterioration of neurological deficiencies documented on a comprehensive neurological investigation or relapse of deficiencies unrelated to other medical diseases (such as fever, infection, or metabolic malfunction). Those who regularly smoked or quit for < 6 months were considered smokers. Alcohol usage was related to current drinking habits or cessation within the past 6 months. The National Institutes of Health Stroke Scale (NIHSS) score was used to determine the degree of neurological deficit at enrollment (16). AIS is described as an abrupt commencement of acute neurological deficiencies validated by computed tomography or magnetic resonance imaging of the brain (14). Individuals with AIS were admitted to the hospital within 3 days following the onset of symptoms. Stroke subtypes were classified according to the Trial of ORG 10172 in Acute Stroke Treatment (TOAST) criteria (17).

### 2.3. Blood pressure measurement and BPV

Systolic blood pressure and DBP readings (casual BP cuffs) were recorded two times each day for 7 successive days (the first time between 6:00 and 8:00 a.m. and the second time between 15:00 and 17:00 p.m., respectively) and checked three times at each time point before calculating the mean. We recorded the BP in the right arm or non-paretic arm with the patient in supine using an automated electronic sphygmomanometer or non-invasive BP monitoring equipment (each patient's blood pressure was measured using the same sphygmomanometer throughout the hospitalization) and recorded the results manually into the patient's medical data according to the clinical regime. The same batch of sphygmomanometers was used for BP measurements. Using the standard deviation (SD) and coefficient of variation (CV) (defined asSD/mean × 100%) of SBP and DBP data within 7 days after stroke onset, we analyzed daily BPV in the acute stage of ischemic stroke (18).

### 2.4. Definition of outcomes

Three months after the onset of symptoms, neurofunctional outcomes were examined using the modified Rankin scale (mRS) (19). Three months following the qualification incident, the clinical data on the outcomes after dismissal were prospectively collected *via* normal clinic visits or through telephone surveys with patients or their caregivers. We divided the functional outcome based on the mRS score: a positive outcome (functional independence) was classified as an mRS score of 0–2 (20), whereas an adverse outcome was classified as moderate to extreme impairment or fatality with an mRS score ≥3, including the recurrence of AIS or intracranial hemorrhage.

### 2.5. Statistical analysis

To optimize the efficiency of statistics and decrease the potential bias induced by the exclusion of variables due to their unavailability, dummy variables were used to indicate missing covariate values. Sensitivity analyses were also performed using a complete-case analysis. To examine continuous variables, findings were displayed as mean ± SD or median (interquartile range). Frequencies or proportions were displayed to examine categorical variables. Logistic regression models were employed to assess the relationship between daily BPV and adverse outcomes.

Both unadjusted and multivariate-adjusted models were used. Variables for adjustments were selected according to their clinical significance and univariate analysis variables with *P* < 0.05. Model 1 was modified for age and sex, while Model 2 was also modified for smoking, alcohol consumption, hypertension, TOAST classification, NIHSS score at enrollment, FPG when admitted, LDL, and neutrophils. Based on Model 2, the mean BP data were added to Model 3. We also used smooth curve fitting (cubic spline smoothing) to determine a non-linear relationship between daily BPV and an adverse outcome. If a non-linear link is discovered, a linear regression model with two piecewise components is employed to estimate the threshold impact and locate the inflection point of the two straight lines (recursive method). To eliminate the deviation resulting from the variation in the degree of daily BPV, a sensitivity analysis was performed to further validate the reliability of the conclusion by changing daily BPV into a categorical variable and calculating the trend P. Additionally, subgroup data were analyzed using the logistic regression model, and subgroup interconnections were examined using likelihood ratio testing (adjustment was performed for clinically significant factors and univariate analysis variables with a *P*-value of 0.05). The R Statistical Program (http://www.R-project.org, The R Foundation) and the Free Statistics analytic platform were used for all the analyses.

## 3. Results

### 3.1. Baseline characteristics of participants

This trial enrolled 633 participants who met the inclusion criteria. The mean age of the trial population was 68 ± 12.1 and 34.4% of the participants were women. At enrollment, the median NIHSS score was 2.5 (interquartile range: 1–5). Of the patients, 7.4% received intravenous thrombolysis, and 6.2% underwent endovascular mechanical thrombectomy. According to the definition of adverse outcomes, 89 patients (14.06%) were described as having adverse outcomes within 3 months following AIS. Among 89 patients with adverse outcomes, 15 died, 13 developed recurrent stroke, 1 had symptomatic intracranial hemorrhage, and others remained disabled. Table 1 presents a comparative summary of the initial attributes. Patients with adverse outcomes were more likely to be older, have a larger percentage of diabetes or AF, have more serious neurological deficits at enrollment, have an increased prevalence of END, and have increased neutrophil counts.

**Table 1:** Comparison of baseline characteristics according to functional status within 3 months.

**Variables**	**Total** **(*n* = 633)**	**Positive outcome** **(*n* = 544)**	**Adverse outcome** **(*n* = 89)**	**p**
Age, Mean ± SD	68.0 ± 12.1	66.8 ± 11.8	75.6 ± 10.9	< 0.001
Gender, *n* (%)				0.009
Male	415 (65.6)	368 (67.6)	47 (52.8)	
Female	218 (34.4)	176 (32.4)	42 (47.2)	
Alcohol consumption, *n* (%)				0.075
No	507 (80.1)	429 (78.9)	78 (87.6)	
Yes	126 (19.9)	115 (21.1)	11 (12.4)	
Smoking, *n* (%)				0.002
No	439 (69.4)	364 (66.9)	75 (84.3)	
Yes	194 (30.6)	180 (33.1)	14 (15.7)	
CHD, *n* (%)				0.114
No	518 (81.8)	451 (82.9)	67 (75.3)	
Yes	115 (18.2)	93 (17.1)	22 (24.7)	
AF, *n* (%)				0.004
No	569 (89.9)	497 (91.4)	72 (80.9)	
Yes	64 (10.1)	47 (8.6)	17 (19.1)	
HLP, *n* (%)				0.956
No	459 (76.1)	395 (76.3)	64 (75.3)	
Yes	144 (23.9)	123 (23.7)	21 (24.7)	
HT, *n* (%)				0.566
No	154 (24.3)	135 (24.8)	19 (21.3)	
Yes	479 (75.7)	409 (75.2)	70 (78.7)	
DM, *n* (%)				0.027
No	349 (55.2)	310 (57.1)	39 (43.8)	
Yes	283 (44.8)	233 (42.9)	50 (56.2)	
IV thrombolysis, *n* (%)				1
No	586 (92.6)	504 (92.6)	82 (92.1)	
Yes	47 (7.4)	40 (7.4)	7 (7.9)	
MT, *n* (%)				0.994
No	594 (93.8)	511 (93.9)	83 (93.3)	
Yes	39 (6.2)	33 (6.1)	6 (6.7)	
**Medical history**				0.067
Antiplatelet use, *n* (%)				
No	517 (81.7)	451 (82.9)	66 (74.2)	
Yes	116 (18.3)	93 (17.1)	23 (25.8)	
Statin use, *n* (%)				0.111
No	537 (84.8)	467 (85.8)	70 (78.7)	
Yes	96 (15.2)	77 (14.2)	19 (21.3)	
Antihypertensive treatment, *n* (%)				0.625
No	273 (43.1)	232 (42.6)	41 (46.1)	
Yes	360 (56.9)	312 (57.4)	48 (53.9)	
Antidiabetic treatment, *n* (%)				0.003
No	440 (69.5)	391(71.9)	49 (55.1)	
Yes	193 (30.5)	153 (28.1)	40 (44.9)	
**Medications during hospitalization**				
Statin use, *n* (%)				0.363
No	24 (3.8)	19 (3.5)	5 (5.6)	
Yes	609 (96.2)	525 (96.5)	84 (94.4)	
Antiplatelet use, *n* (%)				0.004
No	34 (5.4)	23 (4.2)	11 (12.4)	
Yes	599 (94.6)	521 (95.8)	78 (87.6)	
Antihypertensive treatment, *n* (%)				0.048
No	246 (38.9)	203 (37.3)	43 (48.3)	
Yes	387 (61.1)	341 (62.7)	46 (51.7)	
TOAST classification, *n* (%)				< 0.001
SAO (*n*, %)	297 (46.9)	245 (45)	52 (58.4)	
LAA (*n*, %)	250 (39.5)	233 (42.8)	17 (19.1)	
CE (*n*, %)	71 (11.2)	53 (9.7)	18 (20.2)	
Unclassified (*n*, %)	15 (2.4)	13 (2.4)	2 (2.2)	
OCSP classification, *n* (%)				< 0.001
PACI (*n*, %)	297 (46.9)	244 (44.9)	53 (59.6)	
TACI (*n*, %)	19 (3.0)	8 (1.5)	11 (12.4)	
POCI (*n*, %)	137 (21.6)	122 (22.4)	15 (16.9)	
LACI (*n*, %)	180 (28.4)	170 (31.2)	10 (11.2)	
NIHSS score at enrollment	2.5 (1.0, 5.0)	2.0 (1.0, 4.0)	9.0 (5.0, 12.0)	< 0.001
**Median (IQR)**				
mRS score at enrollment	2.0 (1.0, 3.0)	1.0 (1.0, 3.0)	4.0 (4.0, 5.0)	< 0.001
**Median (IQR)**				
END, *n* (%)				< 0.001
No	542 (85.6)	482 (88.6)	60 (67.4)	
Yes	91 (14.4)	62 (11.4)	29 (32.6)	
Neutrophils, Mean ± SD	66.4 ± 10.6	65.8 ± 10.1	70.1 ± 12.6	< 0.001
FPG at enrollment, Mean ± SD	6.4 ± 2.7	6.2 ± 2.4	7.2 ± 3.7	0.001
HDL, Mean ± SD	1.3 ± 0.3	1.3 ± 0.3	1.3 ± 0.3	0.877
LDL, Mean ± SD	2.6 ± 0.9	2.6 ± 0.9	2.4 ± 0.9	0.055

CHD, coronary heart disease; AF, atrial fibrillation; HLP, hyperlipidemia; HT, hypertension; DM, diabetes mellitus; IVT, intravenous thrombolysis; MT, mechanical thrombectomy; TOAST, Trial of Org 10 172 in acute stroke treatment; LAA, large artery atherosclerosis; CE, cardio-embolism; SAO, small artery occlusion; OCSP, Oxfordshire Community Stroke Project; PACI, partial anterior circulation infarction; TACI, total anterior circulation infarction; POCI, posterior circulation infarction; LACI, lacunar infarction; NIHSS, National Institutes of Health Stroke Scale; mRS, modified Rankin scale; END, early neurological deterioration; FPG, fasting plasma glucose; HDL, high-density lipoprotein; LDL, low-density lipoprotein.

### 3.2. Continuous BPV and 3-month neurological functional outcomes

**Figure 2:**
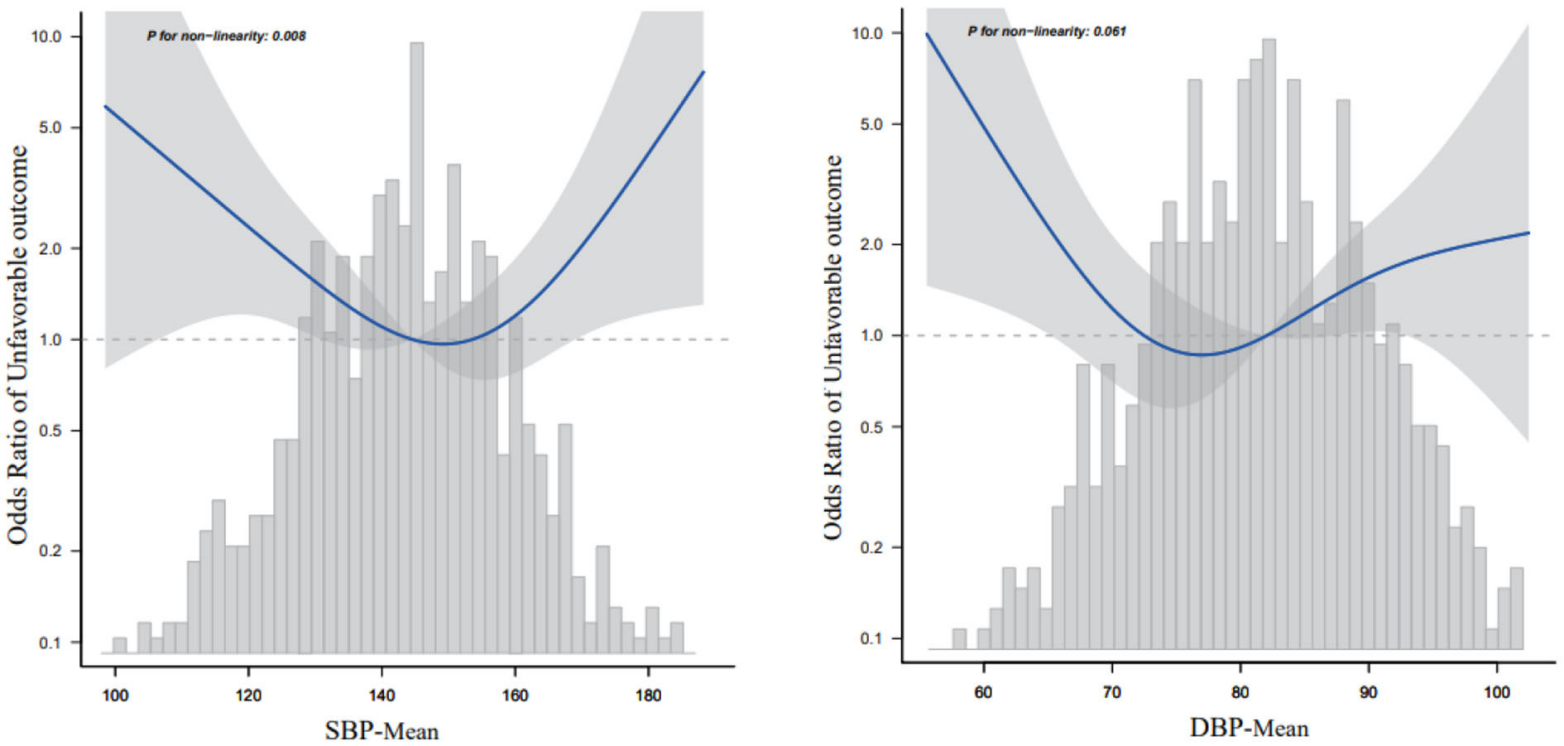
Relationship between mean blood pressure and 3-month adverse outcomes according to the smooth curve-fitting analysis. Adjusted for age, sex, smoking, alcohol consumption, HT, fasting plasma glucose at enrollment, low-density lipoprotein, neutrophils, TOAST classification, NIHSS score at enrollment, and early neurological deterioration. Only 95% of the data are displayed.

Table 2 presents the OR and 95% Cis for daily BPV with adverse outcomes. According to the results in Table 1 and their clinical significance, we included age, sex, smoking, alcohol consumption, HT, FPG at enrollment, LDL, neutrophils, TOAST classification, NIHSS score at enrollment, and END in multiple logistic regression analysis. Independently, every one of each BPV characteristic was incorporated into the logistic models. The SBP-SD, SBP coefficient of variation (SBP-CV), and the DBP-mean were linked to an elevated risk of an adverse outcome at 3 months in the basic model. In a multivariable logistic regression analysis using various logistic regression methods, the correlation between the parameters of BPV and the likelihood of an adverse outcome was extensively investigated. In Model 1, after modifying for sex and age, the SD of BP and the CV of BP both had a positive relationship with an adverse outcome (odds ratio [OR] = 1.1, 95% CI: 1.03–1.16, *P* = 0.002 for SD; OR =1.13, 95% CI: 1.04–1.23, *P* = 0.006 for CV), but not the mean BP (OR =1.01, 95% CI: 0.99–1.02, *P* = 0.378). In Model 2, the significance of the connection between SD or CV of SBP and an adverse outcome persisted, but the association between SBP-mean did not (OR = 1.01, 95% CI: 0.98–1.04, *P* = 0.464). According to the smooth curve-fitting analysis, a U-shaped curve connection was identified between the SBP-mean and an adverse outcome. The curve inflection points of the SBP-mean were within the limits of 152.393–153.631 mmHg (Figure 2). Both SD and the CV of SBP were significantly correlated with an adverse functional outcome in Model 3, following further adjustments for the mean BP (OR = 1.12, 95% CI: 1.01–1.23, *P* = 0.028 for SD; OR = 1.16, 95% CI: 1.01–1.34, *P* = 0.038 for CV) (Table 2).

**Table 2:** Relationship between daily blood pressure variability and adverse outcomes within 3 months in different models.

	**Crude model**		**Model I**		**Model II**		**Model III**	
**Variable**	**OR(95%CI)**	***P*** **-value**	**OR(95%CI)**	***P*** **-value**	**OR(95%CI)**	***P*** **-value**	**OR(95%CI)**	***P*** **-value**
SBP-mean	1.01 (1–1.02)	0.18	1.01 (0.99–1.02)	0.378	1.01 (0.98–1.04)	0.464		
SBP-SD	1.12 (1.06–1.18)	< 0.001	1.1 (1.03–1.16)	0.002	1.12 (1.02–1.23)	0.022	1.12 (1.01–1.23)	0.028
SBP-CV	1.16 (1.07–1.25)	< 0.001	1.13 (1.04–1.23)	0.006	1.15 (1–1.33)	0.045	1.16 (1.01–1.34)	0.038
DBP-mean	0.97 (0.95–0.99)	0.012	1 (0.97–1.02)	0.897	1.02 (0.97–1.06)	0.453		
DBP-SD	1 (0.99–1.02)	0.763	1 (0.98–1.02)	0.761	1.01 (0.98–1.04)	0.404	1.01 (0.98–1.04)	0.63
DBP-CV	1.01 (0.99–1.04)	0.187	1.01 (0.99–1.03)	0.327	1.02 (0.99–1.06)	0.235	1.02 (0.98–1.06)	0.378

Model 1 was adjusted for age and sex.

Model 2 was adjusted for Model 1 plus variables including smoking, alcohol consumption, hypertension, FPG at enrollment, low-density lipoprotein, neutrophils, TOAST classification, NIHSS score at enrollment, and END.

Model 3 included mean BP values based on Model 2.

OR, odds ratio; CI, confidence interval; BP, blood pressure; SBP, systolic blood pressure; DBP, diastolic blood pressure; SD, standard deviation; CV, coefficient of variation.

### 3.3. Categorized BPV and 3-month neurological functional outcomes

The participants were categorized into quartile groups according to various criteria of daily BPV. To apply the multivariable logistic regression analysis, the quartiles with the lowest values were employed as control groups (Table 3). The findings revealed that participants who were in the fourth quartile (SBP-SD: OR = 2.3, 95% [CI]: 1.08–4.9, *P* = 0.031; SBP-CV: OR = 2.31, 95% [CI]: 1.11–4.83, *P* = 0.026) and DBP-SD (OR = 2.13, 95% [CI]: 1.02–4.48, *P* = 0.045; DBP-CV:OR = 2.66, 95%CI 1.28–5.52, *P* = 0.009) had a significantly elevated risk of adverse outcomes following modifying the whole variables including age, sex, smoking, alcohol consumption, hypertension, FPG at enrollment, LDL, neutrophils, TOAST classification, NIHSS score at enrollment, END, and mean BP.

**Table 3:** Odds ratios for adverse outcomes according to quartiles of BP variability.

**Variable**	**n.total**	**n.event%**	**crude.OR(95%CI)**	**Crude.P_value**	**adj.OR(95%CI)**	**adj.P_value**
**SBP-SD**						
Q1	158	15 (9.5)	1(Ref)		1(Ref)	
Q2	158	16 (10.1)	1.07 (0.51–2.26)	0.85	1.15 (0.51–2.61)	0.738
Q3	158	23 (14.6)	1.62 (0.81–3.24)	0.169	1.54 (0.71–3.35)	0.27
Q4	159	35 (22)	2.69 (1.4–5.16)	0.003	2.3 (1.08–4.9)	0.031
Trend.test				0.001		0.017
**SBP-CV**						
Q1	158	15 (9.5)	1(Ref)		1(Ref)	
Q2	158	20 (12.7)	1.38 (0.68–2.81)	0.372	1.16 (0.52–2.54)	0.719
Q3	158	19 (12)	1.3 (0.64–2.67)	0.469	1.23 (0.55–2.74)	0.612
Q4	159	35 (22)	2.69 (1.4–5.16)	0.003	2.31 (1.11–4.83)	0.026
Trend.test				0.003		0.018
**DBP-SD**						
Q1	158	16 (10.1)	1(Ref)		1(Ref)	
Q2	158	17 (10.8)	1.07 (0.52–2.2)	0.854	1.1 (0.5–2.44)	0.805
Q3	158	23 (14.6)	1.51 (0.77–2.99)	0.233	1.43 (0.67–3.07)	0.355
Q4	159	33 (20.8)	2.32 (1.22–4.42)	0.01	2.13 (1.02–4.48)	0.045
Trend.test				0.004		0.029
**DBP-CV**						
Q1	158	16 (10.1)	1(Ref)		1(Ref)	
Q2	158	12 (7.6)	0.73 (0.33–1.6)	0.43	0.71 (0.3–1.69)	0.44
Q3	158	18 (11.4)	1.14 (0.56–2.33)	0.717	1.15 (0.52–2.56)	0.731
Q4	159	43 (27)	3.29 (1.76–6.14)	< 0.001	2.66 (1.28–5.52)	0.009
Trend.test				< 0.001		0.001

Adjusted for age, sex, smoking, alcohol consumption, hypertension, fasting plasma glucose at enrollment, low-density lipoprotein, neutrophils, TOAST classification, NIHSS score at enrollment, END, and mean blood pressure.

BP, blood pressure; Q, quartile; Q1, first quartile; Q2, second quartile; Q3, third quartile; Q4, fourth quartile; OR, odds ratio.

### 3.4. Relationship between mean SBP and 3-month adverse outcomes according to the smooth curve-fitting analysis

Because the SBP-mean was considered a continuous variable in this investigation, restricted cubic spline smoothing curve fitting was employed to examine the non-linear connection between the SBP-mean and 3-month adverse outcomes (Figure 2). After adjusting for all variables including age, sex, smoking, alcohol consumption, hypertension, FPG at enrollment, LDL, neutrophils, TOAST classification, NIHSS score at enrollment, and END, a U-shaped curve association was observed between SBP-mean and 3-month adverse outcomes, and the curve inflection points of SBP-mean were within the limits of 151.032–152.414 mmHg. We discovered a negative correlation between SBP-mean and adverse outcomes on the left side (SBP-mean 151 mmHg) of the inflection point (OR: 0.965, 95% CI: 0.935–0.996, *P* = 0.0275). This was determined by employing a two-stage logistic regression method to compute the saturation impact of the SBP-mean on the occurrence of adverse outcomes based on the smoothing curve and its inflection point. At a mean SBP > 151 mmHg, which is the right side of the inflection point, there was a positive correlation between SBP-mean and adverse outcomes (OR, 1.06; 95% CI: 1–1.123, *P* = 0.0486) (Table 4). Similar trends were observed for the DBP-mean (Figure 2).

**Table 4:** Results of the two piecewise linear regression model.

	**adj.OR(95%CI)**	***P*** **-value**
The inflection point of SBP-mean	151.7 (151,152.4)	
SBP-mean <151 mmHg	0.965 (0.935–0.996)	0.0275
SBP-mean>151 mmHg	1.06 (1–1.123)	0.0486

Adjusted for age, sex, smoking, alcohol consumption, hypertension, fasting plasma glucose at enrollment, low-density lipoprotein, neutrophils, TOAST classification, NIHSS score at enrollment, and END.

### 3.5. Relationship between BPV and 3-month adverse outcomes according to the smooth curve-fitting analysis

**Figure 3:**
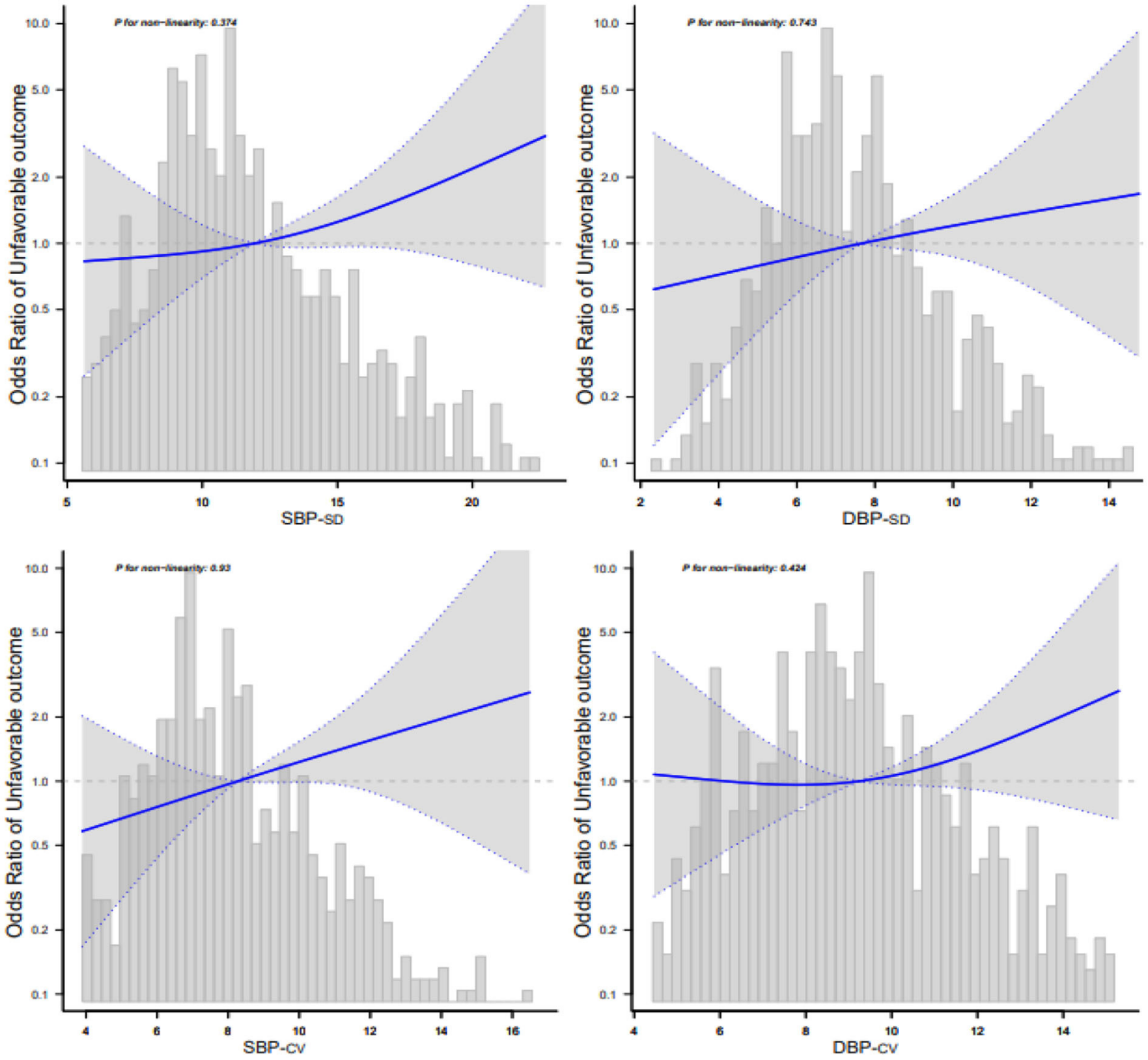
Relationship between blood pressure variability and 3-month adverse outcomes according to the smooth curve-fitting analysis. Adjusted for age, sex, smoking, alcohol consumption, hypertension, fasting plasma glucose at enrollment, low-density lipoprotein, neutrophils, TOAST classification, NIHSS score at enrollment, END, and mean blood pressure. Only 95% of the data are displayed.

We used smooth curve fitting (cubic spline smoothing) to determine a possible non-linear correlation between BPV and adverse outcomes. The results revealed a positive relationship between BPV and adverse outcomes. The risk of adverse outcomes increased with increasing BP-SD or BP-CV (Figure 3).

### 3.6. Subgroup analysis

**Figure 4:**
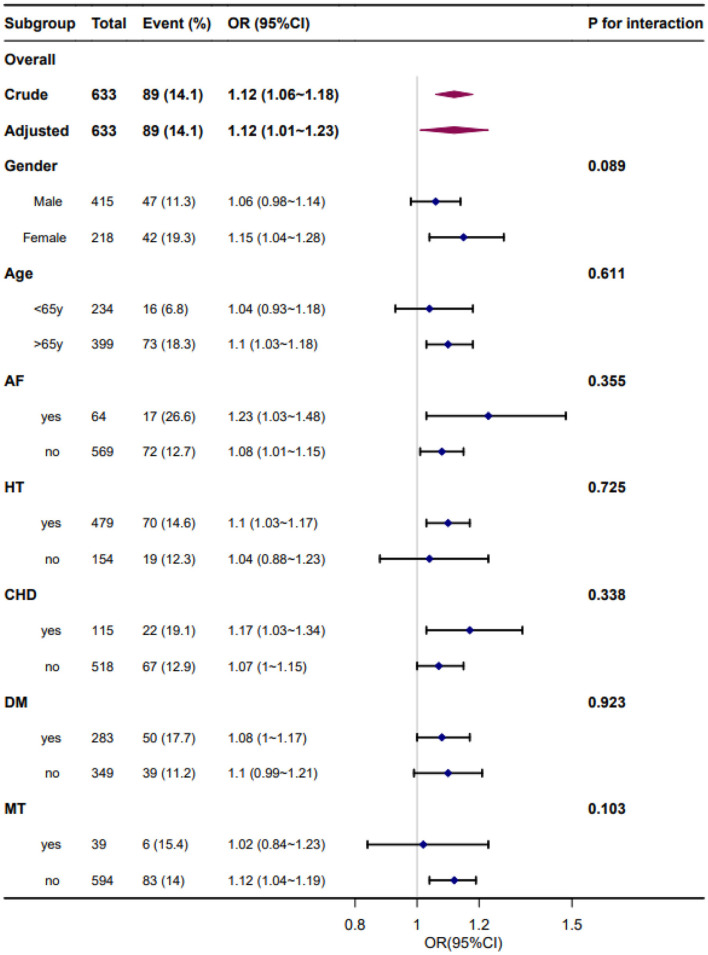
Forest plot of the standard deviation of systolic blood pressure and adverse outcomes by subgroup and interactions. Adjusted for age, sex, smoking, alcohol consumption, hypertension, fasting plasma glucose at enrollment, low-density lipoprotein, neutrophils, TOAST classification, NIHSS score at enrollment, early neurological deterioration, and mean blood pressure. AF, atrial fibrillation; HT, hypertension; CHD, coronary heart disease; DM, diabetes mellitus; MT, mechanical thrombectomy.

**Figure 5:**
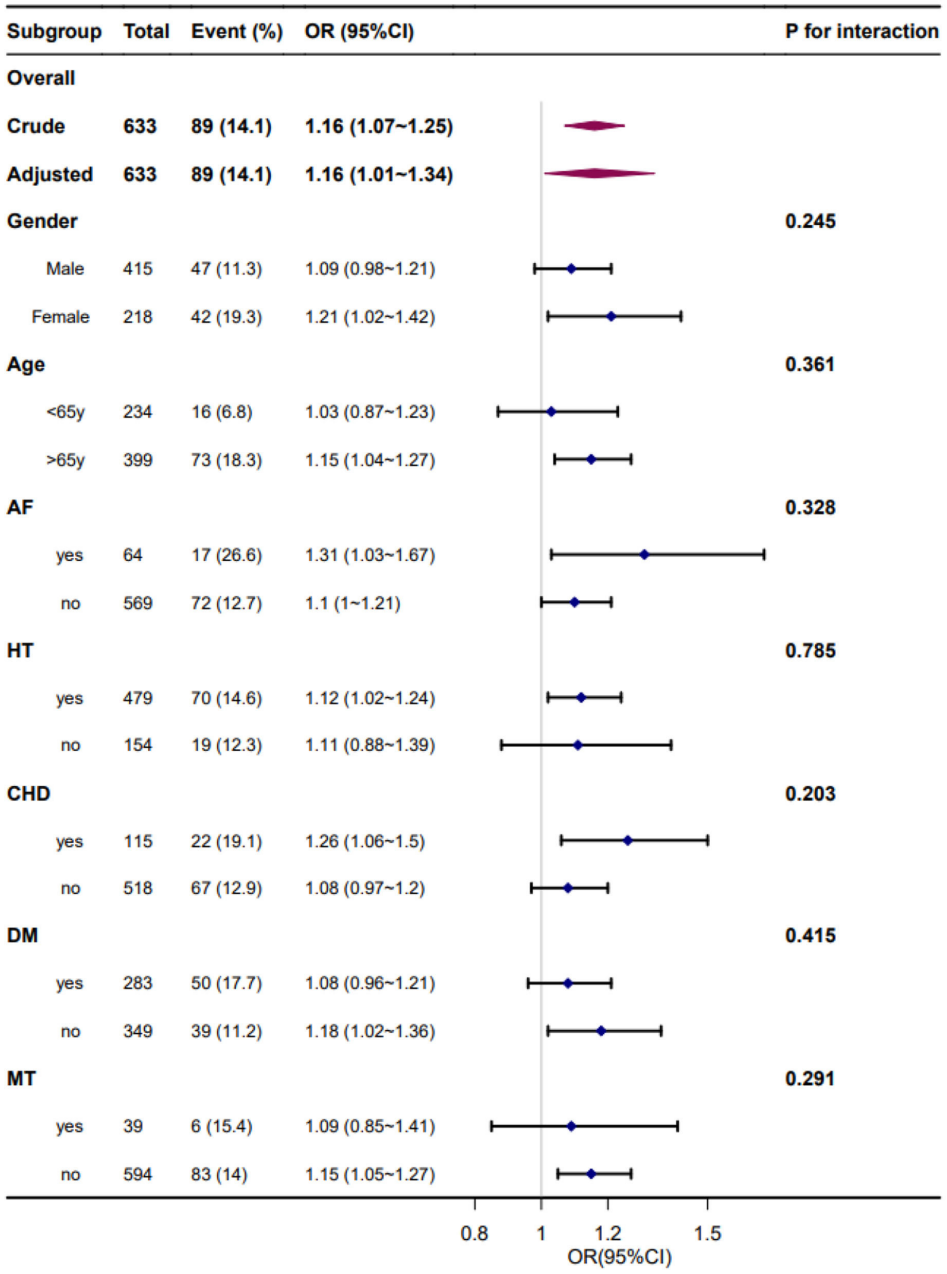
Forest plot of the coefficient variation of systolic blood pressure and adverse outcomes by subgroups and interactions. Adjusted for age, sex, smoking, alcohol consumption, hypertension, fasting plasma glucose at enrollment, low-density lipoprotein, neutrophils, TOAST classification, NIHSS score at enrollment, early neurological deterioration, and mean blood pressure. AF, atrial fibrillation; HT, hypertension; CHD, coronary heart disease; DM, diabetes mellitus; MT, mechanical thrombectomy.

Forest plots of the correlation between SBPV and adverse outcome incidence by subgroups and interactions are presented in Figures 4, 5. The adjustment was performed for age, sex, smoking, alcohol consumption, hypertension, FPG at enrollment, LDL, neutrophils, TOAST classification, NIHSS score at enrollment, END, and mean BP. We found similar associations between all subgroups, and all interactions between subgroups were non-significant for adverse outcomes at a significance level of *P* < 0.05.

## 4. Discussion

According to the findings of our research, an increased daily BP fluctuation, specifically of the SBP in patients with AIS, was associated with a higher risk of an adverse outcome at 3 months, and this risk was independent of BP levels. A relationship between mean BP and adverse outcomes that look like a U- or reverse-J shape may also exist. Recent research has examined the connection between the variability of BP in AIS and outcomes, although the nature of this connection remains unknown.

The findings of our study were consistent with those of other research, which revealed a positive correlation between BPV and an adverse outcome within 3 months in general ischemic stroke, regardless of the mean BP level (7, 8, 21, 22). However, the majority of attention in research done in the past was paid to the impact of short-term changes in BP on the prognosis of AIS or the subacute stage of stroke. Our findings go beyond those of earlier research, which focused solely on the first 24 h or more than 72 h following AIS. Recurring measurements within a short time frame, especially when employing ambulatory BP measurement for the full 24 h that involves several inflations and deflations of the cutoff, affected the patients' overall level of restful sleeping and altered their natural circadian pattern of BP, which may have affected the precision of BPV measurements (23). The conclusion of the research that deals with BPV 72 h after stroke onset and functional outcome (21, 22) was consistent with our study but mainly focused on the subacute stage of AIS. Two previous studies concerning daily BPV validated and improved these findings (24, 25). Wang et al. (24) attempted to explore the association between mid-term BPV within 7 days of onset and prognosis in patients with AIS, and they disclosed consistent observations. In their study, they aimed to determine a possible connection between the two. However, our investigations are distinct in several aspects from one another. In that experiment, BP was monitored every 4 h from day 1 to day 7 (the specific time points were 1 a.m., 5 a.m., 9 a.m., 1 p.m., 5 p.m., and 9 p.m.); however, the average BP throughout the course of the 7 days was not modified or reported at any point. Yang et al. (25) demonstrated that an elevated daily BPV of SBP or DBP in patients with AIS was linked to an increased risk of adverse outcomes within 3 months, regardless of BP readings. However, our conclusion regarding the relationship between mean BP in the 7 days and functional outcomes was different. In their study, a significant dose-response correlation between mean BP and the risk of adverse outcomes was identified. However, the results of our research demonstrated either a U-shaped or reverse J-shaped relationship between mean BP and the risk of adverse outcomes.

Additionally, our study observed that mean BP displayed either a U-shaped or reverse J-shaped connection with an adverse outcome after 3 months. Both extremes, namely, <110 mmHg and >180 mmHg, would be connected to an adverse outcome for the mean SBP. This characteristic is consistent with the belief that a low or high BP in patients who have had a stroke increases the probability of a bad prognosis (22, 26).

Our study also included subgroup analyses to investigate the association between daily BPV and functional stroke outcomes in specific populations. In the subgroup analyses, the association between daily BPV and adverse outcomes at 3 months was stronger in patients with hypertension, AF, and coronary heart disease. This finding underscores the importance of paying more attention to elevated BPV levels in patients with hypertension, AF, and coronary heart disease in the acute stage of stroke. These risk factors may also be the cause of increased daily BPV in patients with AIS.

A few different pathways could lead to an adverse outcome due to a higher BPV. Initially, abnormal sympathetic nervous system stimulation following a stroke is rather frequent and could cause fluctuations in BP (27, 28). An overactive sympathetic nervous system causes a surge in pro-inflammatory cytokines, which causes an inflammatory reaction that typically results in a permeable blood-brain barrier (29–31). Ischemia on its own can trigger a vigorous inflammatory reaction, which contributes to the destruction of the cerebral vasculature and puts the patient at risk for a negative prognosis. Second, BPV may influence hemodynamic conditions through the development of AIS. An increased BPV may worsen the hypoperfusion of the human brain, which may result in the extension of a lesion or even relapse of a stroke as well as other vascular disorders. An increased BPV could be associated with over-perfusion of the delicate ischemic nerves, which may lead to encephala edema, transformation hemorrhage, or other vascular disorders. In conclusion, the under- or over-perfusion of delicate ischemic neurons, which can be induced by elevated BPV, might have an adverse influence on functional outcomes (32).

This study had some limitations, which should be recognized. First, as this was a retrospective study conducted at a single hospital, selection bias might have been present. However, owing to the inclusive nature of our research criteria, the findings have the potential to be generalized. Second, we did not alter the precise information on antihypertensive medications that were collected while the patients were hospitalized. This could have affected both the mean and variability of BP. Although it should be noted that antihypertensive medicine did not contribute to functional outcomes in the baseline characteristics of the participants, the effect of antihypertensive medicines may be reflected in the BP levels itself, and the majority of antihypertensive medicines were empirically initiated prior to or during discharge, which suggests that drug effects were limited in the acute phases by this research characterization (33). Finally, the BP readings for this experiment were obtained from actual clinical practice; thus, the rates of BP readings were lower and even more inconsistent with conventional ambulatory monitoring of BP for 24 h. Additionally, BP was measured in various ways (automated electronic sphygmomanometer or noninvasive BP monitoring), but each patient's blood pressure was measured using the same sphygmomanometer throughout the hospitalization. The findings of the experiment need to be supported by additional prospective research that employs BP measures that are conducted under stricter guidelines.

## 5. Conclusion

Elevated daily BPV of either SBP or DBP in patients with AIS was correlated with an elevated risk of adverse outcomes within 3 months of BP level independence. Additionally, we discovered a U-shaped relationship between the mean BP and adverse clinical outcomes. These findings suggested that BPV should be a risk factor for adverse outcomes after ischemic stroke, which provided new insight into BP management strategy.

## Data availability statement

The raw data supporting the conclusions of this article will be made available by the authors, without undue reservation.

## Ethics statement

The studies involving human participants were reviewed and approved by the Ethics Committee of Jiangsu Province Hospital of Chinese Medicine, Affiliated Hospital of Nanjing University of Chinese Medicine. Written informed consent for participation was not required for this study in accordance with the national legislation and the institutional requirements.

## Author contributions

All authors listed have made a substantial, direct, and intellectual contribution to the work and approved it for publication.
