# Supplementary material for: Daily blood pressure variability in relation to neurological functional outcomes after acute ischemic stroke
Source: Front Neurol. 2023 Jan 9;13:958166. doi: 10.3389/fneur.2022.958166 (PMC9868909; doi:10.3389/fneur.2022.958166)
Supplement: Supplementary file 1 [file Data_Sheet_1.PDF]

Comparison of mean BP and BPV according to functional status within 3 months in hypertensive patients

Variables	Total (n = 479)	positive outcome (n = 409)	adverse outcome (n = 70)	P
SBP-Mean, Mean ± SD	147.3 ± 14.7	146.7 ± 14.0	150.5 ± 18.4	0.046
SBP-SD, Mean ± SD	12.2 ± 4.0	11.9 ± 3.8	13.9 ± 4.6	< 0.001
SBP-CV, Mean ± SD	8.3 ± 2.7	8.1 ± 2.6	9.3 ± 3.2	< 0.001
DBP-Mean, Mean ± SD	83.9 ± 10.0	84.2 ± 10.2	82.3 ± 9.0	0.15
DBP-SD, Mean ± SD	8.6 ± 12.4	8.6 ± 13.4	8.7 ± 2.8	0.946
DBP-CV, Mean ± SD	10.0 ± 8.6	9.8 ± 9.2	10.8 ± 3.6	0.411
