# Supplementary material for: Daily blood pressure variability in relation to neurological functional outcomes after acute ischemic stroke
Source: Front Neurol. 2023 Jan 9;13:958166. doi: 10.3389/fneur.2022.958166 (PMC9868909; doi:10.3389/fneur.2022.958166)
Supplement: Supplementary file 2 [file Table_1.DOCX]

Association between SBP-mean and unfavorable outcomes at 3 months

|  | n.total | n.event_% | crude.OR_95CI | crude.P_ value | adj.OR_95CI | adj.P_ value |
| --- | --- | --- | --- | --- | --- | --- |
| SBP-Mean  140-160mmHg | 294 | 31 (10.5) | 1(Ref) |  | 1(Ref) |  |
| ≤140 mmHg | 235 | 35 (14.9) | 1.48 (0.89~2.49) | 0.134 | 1.95 (1.09~3.49) | 0.025 |
| ≥160 mmHg | 104 | 23 (22.1) | 2.41 (1.33~4.36) | 0.004 | 2.46 (1.26~4.79) | 0.008 |
| Trend.test | 633 | 89 (14.1) | 1.18 (0.86~1.62) | 0.296 | 1.02 (0.71~1.48) | 0.897 |

adjusted for age, gender smoking, alcohol consumption, hypertension, fasting plasma glucose (FPG) at admission, low density lipoprotein, neutrophils, TOASAT classification, NIHSS score at admission, END and mean blood pressure
